# The association of birth weight and postnatal growth with energy intake and eating behavior at 5 years of age – a birth cohort study

**DOI:** 10.1186/s12966-016-0335-4

**Published:** 2016-02-04

**Authors:** Arend W. van Deutekom, Mai J. M. Chinapaw, Tanja G. M. Vrijkotte, Reinoud J. B. J. Gemke

**Affiliations:** Department of Pediatrics, EMGO Institute for Health & Care Research, Institute for Cardiovascular Research VU, VU University Medical Center, Amsterdam, The Netherlands; Department of Public and Occupational Health, EMGO institute for Health & Care Research, VU University Medical Center, P.O. Box 7057, 1007 MB Amsterdam, The Netherlands; Department of Public Health, Academic Medical Centre, University of Amsterdam, Amsterdam, The Netherlands

**Keywords:** Birth weight, Postnatal growth, Developmental origins of health and disease, Childhood obesity, Energy intake, Eating behavior

## Abstract

**Background:**

Low and high birth weight and accelerated postnatal weight gain are associated with an increased risk of obesity. Perinatal effects on energy intake and eating behavior have been proposed as underlying mechanisms. This study aimed to examine the independent associations of birth weight and postnatal weight and height gain with childhood energy intake and satiety response.

**Methods:**

In a birth cohort study, we used data from 2227 children (52 % male), mean age 5.6 (±0.4) years. Mean daily energy intake and satiety response were parent-reported through validated questionnaires. Exposures were birth weight z-score and conditional weight and height gain between 0–1, 1–3, 3–6, 6–12 months and 12 months to 5 years. Conditional weight and height are residuals of current weight and height regressed on prior growth data, to represent deviations from expected growth. Analyses were adjusted for a set of potential confounding variables.

**Results:**

Conditional weight gain between 1–3, 3–6 months and 12 months to 5 years was significantly associated with energy intake, with 29.7 (95 %-CI: 4.6; 54.8), 24.0 (1.8; 46.1) and 79.5 (29.4; 129.7) kcal/day more intake for each Z-score conditional weight gain between 1–3, 3–6 months and 12 months to 5 years, respectively. Conditional height gain between 0–1, 1–3 months and 12 months to 5 years was negatively associated with energy intake (β: −42.0 [66.6; –17.4] for 0–1 months, −35.1 [−58.4; −11.8] for 1–3 months and −37.4 [−72.4; −2.3] for 12 months to 5 years). Conditional weight gain in all periods was negatively associated with satiety response, with effect sizes from − 0.03 (−0.06; −0.002) in early infancy to −0.12 (−0.19; −0.06) in childhood. Birth weight was not associated with energy intake or satiety response.

**Conclusions:**

Our findings suggest that accelerated infant and childhood weight gain are associated with increased energy intake and diminished satiety response at 5 years. Accelerated height gain seems to be beneficial for childhood energy intake. This perinatal ‘programming’ of energy intake and eating behavior provide a potential mechanism linking early life influences with later obesity and cardiovascular disease.

**Electronic supplementary material:**

The online version of this article (doi:10.1186/s12966-016-0335-4) contains supplementary material, which is available to authorized users.

## Background

The alarming rise of childhood obesity world-wide has been identified by the WHO as one of the most serious public health challenges of the 21st century [1]. Secular trends toward increased intake of energy-dense foods and decreased levels of physical activity are recognized as attributable factors to this obesity epidemic, but more recently the perinatal environment has been suggested to play a role. This is based on the observations of numerous population-based cohort studies showing that both low and high birth weight are associated with increased obesity risk [2]. This risk seems to be enhanced when followed by accelerated postnatal weight gain [3], while height gain might protect against subsequent obesity [4]. A proposed explanation for the association of birth weight and postnatal growth with later obesity is through the concept of ‘Developmental Origins of Health and Disease’, which states that environmental cues during critical periods of life elicit predictive adaptive responses that shape tissue development and metabolic pathways, thereby permanently affecting later health and disease risk [5]. However, the mechanisms by which the perinatal environment influence obesity risk, the relative effects of weight and height gains and which period of postnatal growth has the greatest impact on later health are not well established.

There is clear evidence that nutritional insults during prenatal or early postnatal life can impact hypothalamic development and function, with lasting effects on feeding and metabolism [6]. Therefore, one possible mechanism by which birth weight and direct postnatal growth influence the risk of obesity is that the perinatal environment alters the function of central regulatory mechanisms, including appetite regulation. Animal models were the first to show adult hyperphagia [7–9] and altered feeding behavior [10] in low birth weight offspring, especially when low birth weight was followed by rapid postnatal weight gain. A more recent meta-analysis, however, concluded that there is little effect of prenatal caloric restriction on the programming of appetite in rats, and suggested that the critical window for altering feeding behavior might be located after birth [11]. In humans, only a few studies investigated the early-life effects on later energy intake. Two studies showed that prenatal exposure to the Dutch Famine was associated with increased energy intake at middle age [12, 13], while one study from Brazil observed altered feeding preferences in young women born with low birth weight [14]. However, these studies have been criticized as models of mild nutritional deprivation. In the Brazilian cohort, altered feeding preference was restricted to severely growth impaired individuals, and the Dutch Famine cohorts were exposed to environmental stress (war), besides malnutrition. These stress exposures could also affect behavior, including eating behavior, in later life [15]. In addition, the subjects of most adult cohorts already show an increased rate of obesity and metabolic sequelae, and these metabolic changes may by itself influence eating behavior. Therefore, it has been noted that differences in energy intake and eating behavior need to be assessed before these metabolic sequelae become apparent, i.e., in childhood [16]. The effects of postnatal growth on later energy intake remain to be addressed.

The aim of the present study was to assess the independent associations of birth weight, postnatal weight gain and postnatal height gain with mean daily energy intake and eating behavior at 5 years of age. In addition, we assessed whether critical postnatal growth periods exist for the association of growth with energy intake and eating behavior by exploring the associations of discrete time intervals in infancy and childhood with these outcomes. We hypothesized that children with low birth weight and subsequent accelerated weight gain, especially in early infancy, have a higher energy intake and a decreased satiety response at 5 years of age.

## Methods

### Setting

The subjects are participants in the Amsterdam Born Children and their Development (ABCD) cohort, originally consisting of 8266 pregnant female residents of Amsterdam and their offspring. Details of the study, including its rationale, background and measurements, are described in previous publications [17]. A flowchart of the sampling procedure and attrition rates is available as supplementary figure on the study website. Briefly, the women enrolled in this study completed questionnaires during and after their pregnancy covering sociodemographic data, lifestyle, dietary habits and psychosocial factors, conventional medical and obstetric history, and perinatal details. Of the 6735 women who gave permission for follow-up of their child, 6575 mothers also consented to follow up of their child’s growth data. Of these children, 3321 children completed a ‘health check’ at 5 years of age, at which data on growth and body composition were collected, amongst others. In addition, mothers completed questionnaires about the child’s health, development and behavior, and food consumption and eating behaviors.

Written informed consent was obtained from each mother at enrolment and at the age 5 health check. Ethical approval was granted by the Amsterdam Medical Center’s Ethics Committee, and all procedures complied with the ethical standards of the Helsinki Declaration [18].

### Assessment of energy intake and eating behavior

Mean daily energy intake and eating behavior of the children were assessed by the Food Frequency Questionnaire (FFQ) and Child Eating Behavior Questionnaire (CEBQ), respectively, completed by the mothers when the children were 5 years of age.

The FFQ approximates mean daily dietary intake based on reported consumption of 71 food items [19]. The questionnaire is developed for Dutch children based on data from the third Dutch National Food Consumption Survey, and validated in children aged 4–6 years [20]. For each food item parents indicated their child’s habitual consumption frequency in the previous four weeks by checking one of six frequency categories ranging from ‘never’ to ‘6–7 days a week’. Portion sizes were specified in natural units (e.g., one apple), common household measures (e.g., one glass of milk) or grams. The average daily intake of macro- and micronutrients was calculated by multiplying the reported daily intake of each food by its nutrient content, according to the Netherlands Food Composition Table NEVO 2001 [21]. Although a comprehensive diet analysis was performed, only the primary variable of interest (i.e., mean daily energy intake) was included in the present statistical analysis.

The CEBQ is a parent-report questionnaire that measures eight dimensions of eating style in children (food responsiveness, enjoyment of food, satiety responsiveness, slowness in eating, fussiness, emotional overeating, emotional undereating, and desire to drink) [22]. The 35 items are rated on a 5-point scale, ranging from ‘never’ to ‘always’. For this study, the ‘Satiety Response’ dimension was used as the outcome measure, as this represents the ability of a child to reduce food intake to compensate for prior foods/snacks to regulate its energy intake, and thereby is of primary interest for this study. This dimension is a composite measure that averages the scores of the following items: “My child has a big appetite”, “My child leaves food on his/her plate at the end of the meal”, “My child gets full before his/her meal is finished”; “My child gets full easily” and “My child cannot eat a meal if he/she has had a snack just before”. A lower score on this dimension reflects a lower satiety response and thus a more disadvantageous eating behavior. The Cronbach’s alpha for this dimension was 0.80.

### Growth data

Child’s sex, birth weight and gestational age at birth were extracted from records from the regional vaccination register (Entadministratie); the office that also performs screening for inborn errors of metabolism in the first week of life. From birth to 12 months of age, child weight and height were ascertained at on average 8 time points as part of the regular preventive Child Health Care in the Netherlands, performed by qualified nurses and physicians.

At age 5, at the ABCD health check, weight and height were measured by a team of trained researchers according to standard protocols using a Leicester portable height measure (Seca, Birmingham, UK) and a Marsden weighing scale (model MS-4102, Rotherham, UK), respectively. Children were dressed in light clothing during these measurements.

Birth weight and subsequent height and weight at 1, 3, 6 and 12 months and 5 years of age were used in the analysis to reflect pre- and postnatal growth. Since most children had no measurements on these exact ages, weight and height data were interpolated to the exact ages of 1, 3, 6 and 12 months by interpolation using individual weight curves. Prior to interpolation, acceptable age windows were defined, which were 0–2, 2–4, 4–8 and 9–15 months for interpolation at 1, 3, 6 and 12 months, respectively. Only children for whom growth data in all acceptable age windows were available as well as birth weight and 5 year measurements were used in the analysis (*n* = 2533). Birth height was largely missing in our dataset, and therefore not used in the analyses. Data on weight and height were converted to sex-specific weight-for-age and height-for-age Z-scores, respectively, based on the weight and height distributions of the children with complete anthropometric data at all selected ages. Using these Z-scores, we derived growth between successive time points by calculating conditional weight and conditional height variables. The conditional weight and height values are defined as the difference between observed and predicted body size, are uncorrelated by design and are therefore widely used to allow for the high statistical correlation of subsequent weight and height measures at each age [23, 24]. Conditional weight was calculated as the residual from a linear regression of weight at a given age on all prior weights and heights and current height. Conditional height was calculated as the residual from a linear regression of height at a given age on all prior weights and heights but not current weight. The conditional value is thus an independent measure of the deviation in an individual’s observed size from its expected size given all prior growth values. A positive conditional value indicates growing faster than expected given prior size.

### Assessment of potential confounding variables

Several variables potentially related to birth weight, infant growth and/or eating behavior, such as socioeconomic status (SES), ethnicity, maternal and paternal body mass index (BMI), smoking during pregnancy, duration of exclusive breastfeeding, childhood screen time and childhood physical activity (PA) level were considered as potential confounders [25]. SES was based on attained maternal educational level and parents' BMI on self-reported height and weight, collected by questionnaire when the children were 5 years of age. Childhood screen time was based on parents’ report of the duration that their child spent watching TV or used a computer or console, scored on a 7-point scale ranging from ‘(almost) never’ to ‘5 h/day or more’. PA level was based on parents’ responses to questions on duration of playing outside in summer and winter for weekdays and weekend days separately (0–4 h/day) and sports participation (0–4 h/week), which were averaged to produce a PA score (range 0-4) of their child.

### Data analysis

Univariate comparisons of basic demographic characteristics and potential confounding factors between the children included in this analysis and those excluded because of insufficient growth data were performed with use of the Student’s *t*-test for continuous variables and the *χ*^2^ test for discrete variables. Descriptive statistics are given as means and SD for continuous data and as frequency distributions for categorical data, unless otherwise stated.

A multivariable linear regression analysis was conducted to assess the association of birth weight, conditional weight variables and conditional height variables with mean daily energy intake and satiety response at age 5. This analysis was adjusted for a set of potential confounding variables: sex, gestational age, ethnicity, maternal and paternal BMI, socioeconomic status, smoking during pregnancy, duration of exclusive breastfeeding, current age, height and BMI, screen time and PA level. We present both the unstandardized and the standardized regression coefficients, to provide a better estimate of the magnitude of the association. The distribution of the outcome variables and their residuals was checked for normality and transformations were not deemed necessary.

Because of potential sex differences in the developmental origins of obesity [16], effect modification by sex was assessed by addition of interaction terms.

In a sensitivity analysis, we further assessed how associations found in the primary analyses were influenced or driven by extreme values. Therefore, the analyses were redone excluding the subjects with mean daily energy intakes more than +2SD above the mean or less than -2SD below the mean. In addition, because children born preterm (<37 weeks’ gestation) and/or low birth weight (birth weight < -2SD) have substantially different postnatal growth trajectories than children born appropriate-for-gestational age and at term [26], we conducted a sensitivity analysis of the original models excluding these subjects.

Statistical analyses were performed using SPSS Statistics for Windows, Version 17.0. The level of statistical significance was set at 0.05, except for the interaction terms, for which a *P*-value less than 0.10 was considered significant.

## Results

### Descriptive statistics

Two thousand two hundred twenty-seven children of the total ABCD cohort had complete data on growth, energy intake and eating behavior available and were included in the final analysis (see Additional file 1: Figure S1). Relevant baseline characteristics of these children, and a non-response analysis of the remainder of the cohort, are presented in Table 1. Sex-specific growth and outcome variables are presented in Table 2. The parent-reported mean daily energy intake in our cohort ranged from 466 to 4055 kcal/day. This wide distribution was mainly due to a few extreme values, as 98 % of the children had an energy intake between 863 and 2537 kcal/day. Further information about the distribution of the mean daily energy intake is presented in a histogram available as supplementary figure S1 at the *International Journal of Behavioral Nutrition and Physical Activity*‘s website.

**Table 1:** Characteristics of the study subjects and remainder of the ABCD cohort

	Study subjects	Remainder of the cohort	*P* value*
n	2,227	2,904	
Family characteristics			
Ethnicity (%)			<0.01
Dutch	78.7	55.3	
Surinamese	2.4	6.7	
Turkish	1.9	5.0	
Moroccan	3.8	8.6	
Other	13.1	24.4	
Maternal BMI in kg/m^2^	23.5 (4.0)	23.7 (4.1)	0.18
Paternal BMI in kg/m^2^	24.8 (3.0)	25.2 (3.4)	<0.01
Parental SES (%)			<0.01
Low	3.9	6.8	
Mid	26.7	34.5	
High	69.4	58.8	
Maternal smoking during pregnancy (% yes)	6.1	7.5	0.03
Duration of exclusive breastfeeding in weeks [median, IQR]	12.0 (20.0)	8.0 (14.0)	<0.01
Subject characteristics - Birth			
Sex (% male)	51.3	49.1	0.11
Gestational age in weeks	40.1	39.5	<0.01
Birth weight in grammes	3570 (501)	3377 (606)	<0.01
Subject characteristics – Questionnaire data			
Age at FFQ in years	5.7 (0.5)	5.8 (0.5)	<0.01
Mean energy intake in kcal/day	1530 (338)	1554 (372)	<0.01
Age at CEBQ in years	5.1 (0.2)	5.3 (0.4)	<0.01
Satiety Response subscore	2.4 (0.5)	2.4 (0.5)	0.25
Screen time in hours/day	1.37 (0.97)	1.56 (1.15)	<0.01
PA score (range 0–4)	1.30 (0.60)	1.22 (0.61)	<0.01

Descriptive statistics for the study participants and the remainder of the ABCD cohort. Data presented as means and SD in parenthesis unless otherwise stated

*Abbreviations*: *BMI* Body mass index, *SES* Socioeconomic status, *FFQ* Food Frequency Questionnaire, *CEBQ* Child Eating Behavior Questionnaire, *PA* Physical activity

*Student’s *t*-test for continuous variables, Pearson *χ*2 for dichotomous variable

**Table 2:** Descriptive growth and outcome data of the study subjects

	Boys	Girls
N	1147	1080
Growth characteristics		
Weight in kilogrammes		
Birth	3.61 (0.50)	3.48 (0.46)
1 month	4.57 (0.55)	4.28 (0.49)
3 months	6.39 (0.68)	5.83 (0.62)
6 months	8.09 (0.82)	7.45 (0.76)
12 months	10.2 (1.0)	9.5 (1.0)
5 years	21.2 (3.0)	21.0 (3.4)
Height in centimetres		
1 months	55.2 (2.0)	54.1 (2.0)
3 months	62.0 (2.0)	60.4 (1.9)
6 months	68.4 (2.1)	66.6 (2.0)
12 months	76.5 (2.4)	74.9 (2.3)
5 years	116.8 (5.5)	116.1 (5.7)
BMI at 5 years in kg/m^2^	15.5 (1.3)	15.5 (1.6)
Primary outcome measures		
Mean energy intake in kcal/day	1585 (339)	1583 (325)
Satiety Response subscore	2.3 (0.5)	2.4 (0.5)

Sex-specific descriptive growth and outcome data for the study participants. Data presented as means and SD in parenthesis

### Primary results

Table 3 presents the results of the regression analyses of the association of birth weight, conditional weight and conditional height with mean daily energy intake and satiety response at age 5, fully adjusted for a range of confounders. Conditional weight gain between 1 and 3, and then 3 and 6 months, was positively associated with energy intake, with a 29.7 kcal/day (95 %-CI: 4.6; 54.8. *P* = 0.02) and a 24.0 kcal/day (95 %-CI: 1.8; 46.1. *P* = 0.03) higher intake at age 5 for every Z-score conditional weight gain between 1–3 months and 3–6 months, respectively. The magnitude of the association between childhood conditional weight gain (i.e., between 12 months and 5 years) and energy intake was approximately 3 times greater (β = 79.5. 95 %-CI: 29.4; 129.7. *P* = 0.002). Conditional height gain in early infancy was inversely associated with energy intake: for every Z-score increase in conditional height between 0–1 and 1–3 months, mean daily energy intake was 42.0 kcal (95 %-CI: −66.6; −17.4. *P* < 0.001) and 35.1 kcal (95 %-CI: −58.4; −11.8. *P* = 0.003) lower at age 5. In addition, there was an inverse association of childhood conditional height gain with energy intake of similar magnitude.

**Table 3:** Association of birth weight, conditional weight and conditional height with mean daily energy intake and satiety response

	Energy intake (kcal/day) B (95 %-CI)	Standard. Beta (95 %-CI)	*P*-value	Satiety Response (score) B (95 %-CI)	Standard. Beta (95 %-CI)	*P*-value
Weight						
Birth weight	−7.1 (−30.9; 16.7)	−0.02 (−0.09; 0.05)	0.56	−0.01 (−0.04; 0.02)	−0.02 (−0.08; 0.04)	0.51
Conditional weight 0–1 month	4.5 (−16.1; 25.2)	0.01 (−0.05; 0.08)	0.67	−0.03 (−0.06; −0.00)	−0.06 (−0.12; −0.00)	0.03
Conditional weight 1–3 months	29.7 (4.6; 54.8)	0.09 (0.01; 0.16)	0.02	−0.05 (−0.08; −0.01)	−0.08 (−0.15; −0.01)	0.006
Conditional weight 3–6 months	24.0 (1.8; 46.1)	0.07 (0.01; 0.14)	0.03	−0.04 (−0.07; −0.01)	−0.07 (−0.13; −0.00)	0.01
Conditional weight 6–12 months	8.9 (−14.5; 32.2)	0.03 (−0.04; 0.10)	0.46	−0.07 (−0.10; −0.04)	−0.15 (−0.21; −0.08)	<0.001
Conditional weight 12 months–5 years	79.5 (29.4; 129.7)	0.23 (0.08; 0.37)	0.002	−0.12 (−0.19; −0.06)	−0.23 (−0.37; −0.10)	<0.001
Height						
Conditional height 0–1 month	−42.0 (−66.6; −17.4)	−0.13 (−0.20; −0.05)	<0.001	0.01 (−0.02; 0.04)	0.00 (−0.06; 0.06)	0.48
Conditional height 1–3 months	−35.1 (−58.4; −11.8)	−0.11 (−0.18; −0.04)	0.003	0.00 (−0.03; 0.02)	−0.02 (−0.07; 0.04)	0.94
Conditional height 3–6 months	−9.0 (−29.9; 11.9)	−0.03 (−0.09; 0.03)	0.40	−0.02 (−0.05; 0.00)	−0.06 (−0.11; 0.00)	0.06
Conditional height 6–12 months	−9.4 (−31.4; 12.6)	−0.03 (−0.09; 0.04)	0.40	−0.02 (−0.04; 0.01)	−0.04 (−0.09; 0.02)	0.12
Conditional height 12 months–5 years	−37.4 (−72.4; −2.3)	−0.11 (−0.21; −0.01)	0.04	−0.02 (−0.05; 0.01)	−0.04 (−0.11; 0.03)	0.24

Results of the regression analysis of the association of birth weight, conditional weight and conditional height (all in Z-scores) with mean daily energy intake and satiety response at age 5. The coefficients are presented both in the original units of measurement and in standardized betas. Analysis adjusted for sex, gestational age, ethnicity, maternal and paternal body mass index, socioeconomic status, smoking during pregnancy, duration of exclusive breastfeeding, current age, height and body mass index, screen time and physical activity score at age 5

All conditional weight variables had inverse associations with satiety response, with incremental regression coefficients from early infancy to childhood: from a 0.03 lower satiety response score (95 %-CI: −0.06 ;−0.002. *P* = 0.03) for every Z-score increase in conditional weight in the first month of life to a 0.12 lower score (95 %-CI: −0.19; −0.06. *P* < 0.001) for every Z-score increase in childhood. Conditional height showed no association with satiety response, and birth weight Z-score was associated with neither mean daily energy intake nor satiety response.

We tested for heterogeneity by sex by including an interaction term of sex with each of the independent variables. All these interaction terms were non-significant (all *P* > 0.10, data not shown), indicating that the associations of birth weight, conditional weight and conditional height with mean daily energy intake and satiety response were similar in boys and girls.

### Sensitivity analyses

A sensitivity analysis, with children with reported mean daily energy intake less than -2SD below or more than +2SD above the mean excluded (*n* = 88), were consistent with the results of the primary analysis, with slightly broader confidence intervals reflecting the smaller sample size available for analysis. The results of the analyses were also materially unaltered following the exclusion of children born before 37 weeks gestation (*n* = 54), with a birth weight less than -2SD below the mean (*n* = 61), or both (*n* = 59). The results of both sensitivity analyses are available as supplementary tables at the journal’s website.

## Discussion

In this population-based cohort, we found a higher mean daily energy intake at age 5 in children with conditional weight gain in early infancy (1 to 6 months) and childhood (12 months to 5 years). Conditional height gain in the first 3 months of life or childhood was associated with lower mean daily energy intake at age 5. In addition, conditional weight gain was associated with a lower satiety response, irrespective of the period in which conditional weight gain occurred. Conditional weight gain in childhood had an effect size approximately three times that of conditional weight gain in infancy, both for its association with energy intake and satiety response. These results were adjusted for a range of confounding variables including child’s current BMI, and were not driven by outliers or children born preterm or with low birth weight, as the exclusion of these children produced similar results.

To the best of our knowledge, this is the first study in humans describing associations between postnatal growth and later energy intake. The limited literature on early growth and energy intake solely focuses on birth weight, as a marker of prenatal growth. Two independent publications on the health effects of prenatal exposure to the Dutch famine reported that prenatal undernutrition is associated with a preference for an energy-dense diet [12, 13], and in one publication with a higher energy intake at middle age [13]. In addition, an association of severe intra-uterine growth retardation with unfavorable dietary habits were found in a Brazilian and a Finnish cohort of young adults [14, 27]. However, in the only available publication on the association of birth weight with childhood energy intake, Shultis et al. found no associations of birth weight with childhood energy intake in a subgroup of the ALSPAC birth cohort [28]. Similarly, a recent study in three prospective birth cohorts found generally no effect of birth weight on eating behaviors in childhood [29]. This is in line with the conclusion of our study that birth weight is not associated with energy intake or satiety response in healthy children. These apparent conflicting results could be explained by the severity of nutritional deprivation, with measurable effects on later intake only after severe intrauterine growth restriction, or the additive impact of other environmental (war) stressors in the famine cohorts. Another possible explanation arises from the observation that associations of birth weight with energy intake and eating behavior are consistently found in adult cohorts, while studies in children, like ours, found no such association. Low birth weight is associated with features of the metabolic syndrome, especially in adulthood [30], which may secondarily influence dietary habits through an altered secretion of adipokines that control energy balance [31, 32]. Altered adult energy intake may thereby be a direct effect of the metabolic syndrome associated with low birth weight, instead of a mediating link in the pathway of low birth weight to obesity.

Our findings suggest that postnatal growth is associated with childhood energy intake, with effect sizes ranging from 24 kcal/day in early infancy to 80 kcal/day in childhood for every Z-score conditional weight gain. In contrast, conditional height gain during infancy or childhood is associated with lower energy intake. This is in line with other studies, showing detrimental effects of accelerated weight gain on later body composition [33] and obesity risk [34], while accelerated height gain is associated with a more healthy body composition characterized by an increase in fat-free mass [35].

Although the effect size of accelerated conditional weight gain may appear small, when considering food consumption even small but chronic excesses in intake could result in a positive energy imbalance resulting in excessive weight gain. For instance, a mathematical model applied to the population of the United States concluded that the obesity epidemic could be explained by an average energy imbalance between intake and expenditure of about 10 kcal/day [36]. In addition, it was estimated that the gradual weight gain of the population of 0.5–1 kg a year over the last two decades can be accounted for by a positive energy imbalance of 15 kcal/day [37]. Therefore, the observed differences in energy intake associated with conditional weight gain could contribute to the increased adiposity risk associated with increased early life growth and hence merit public health attention.

We only found associations of conditional weight and height gain with energy intake for the period of early infancy (first 6 months) and early childhood (1 to 5 years). Although having a different outcome variable, these ‘critical periods’ for the development of childhood energy intake in our study rather consistently resemble the periods in which weight gain is associated with later body composition. Two studies independently reported that rapid weight gain in early infancy (0–6 months) and early childhood (2/3–6 years), but not in the in-between period, were associated with later body composition [38, 39]. In both studies it was suggested that these periods resemble two different critical periods for the developmental origins of obesity, with different underlying mechanisms leading to these associations. In early infancy, the development of hypothalamic centers responsible for energy balance are susceptible to environmental cues. For instance, in animal models, perinatal malnutrition was characterized by the induction of central leptin resistance and changes in hypothalamic circuitry, with life-long effects on food intake, energy expenditure and metabolic regulation [8, 40]. In childhood, accelerated weight gain might influence energy intake by the concept of ‘adiposity rebound’: the rise in BMI after an initial drop that occurs between age 2 and 5. An early onset of this phenomenon is associated with an increased risk of later obesity, independent of other risk factors, and empirical evidence suggests that suboptimal perinatal growth advances the timing of adiposity rebound [41]. Moreover, the age of adiposity rebound is considered of critical importance to the programming of energy balance [42]. Conditional weight gain between 1 and 5 years in our study might thereby reflect an early rise in BMI in the context of an early adiposity rebound, through which eating behaviors and energy intake may be influenced.

Studies are increasingly demonstrating that early life fat accretion, as opposed to a gain in fat-free mass, is implicated in the induction of adult diseases. In our study, infant and childhood weight gain adjusted for height represented a global index of fat accretion. This is common practice in epidemiological studies, because height-adjusted weight gain is consistently associated with increased fat deposition [43, 44], and an increased risk of subsequent obesity [45]. Nonetheless, it represents a very crude index of fat mass, and weight gain has been associated with a gain in fat-free mass as well [38]. Similarly non-specific are birth weight as a marker for prenatal growth and height gain as a marker for fat-free mass. The ABCD study has insufficient longitudinal data based on higher-quality body composition techniques, such as bioelectrical impedance analysis (BIA) and dual energy X-ray absorptiometry (DXA) to address this issue. This implies that future studies of early influences on energy intake or eating behavior should adopt high-quality body composition techniques to assess which body component is critical for the observed effect. However, because in most countries postnatal weight and height is routinely measured on multiple occasions in every child, in contrast to BIA or DXA, its association with obesogenic behaviors has more potential importance for clinical practice and public health, as it allows such growth to be used as a predictor of later risk.

### Strengths and weaknesses

A major strength of the current study is the use of a large cohort of apparently healthy boys and girls, with detailed objectively assessed growth data obtained from reliable records. This data is prospectively collected from birth to childhood, which enables exploration of a longitudinal relationship. In addition, we were able to adjust for many known confounders, such as parental BMI and smoking during pregnancy, and to adjust for height, BMI, screen time and PA, which are known to influence energy intake independent of early growth.

A further strength is the use of conditional growth variables to model growth during discrete time intervals in our cohort. It has been argued that this approach allows for the high correlation of height and size measures, at least partially resolving the problem of collinearity in traditional multiple regression analysis [23]. Our growth measures are, by design, uncorrelated with each other, and the use of Z-scores permits direct comparison of effect sizes across ages.

Several issues in the design and implementation of the study are worth considering. First, we only included children of the ABCD cohort whose weight and height could be estimated for each relevant time point. This reduced the number of eligible children to 2,227, but increased the accuracy of the data analyzed. The accuracy of the estimates depends on accurate measurements of body size and a correct modeling of growth in the ABCD cohort. Measurement errors, outliers and incorrect assumptions of the growth model may potentially impair the accuracy of the estimate. Participation rates were higher among children from higher SES groups and of Dutch descent, so these children were overrepresented in our analysis. Selection bias would be expected to affect the results only if the association of early growth with energy intake and satiety response was different in included children compared with the remainder of the cohort. This is unlikely but cannot be excluded.

Second, the validity of energy intake reporting by the FFQ may be challenged, based on the observation that energy intake is systematically misreported in validation studies of energy intake against objectively measured energy expenditure [46]. In addition, accurate parental assessment of their child’s energy intake is hindered by the child’s out-of-home food intake [47]. However, a review of validation studies in children concluded that parent-reported energy intake was valid in younger children when compared to doubly-labelled water measured energy expenditure [48]. More specifically to our study, energy intake derived from the FFQ was considered in good agreement to energy expenditure in thirty Dutch children aged 4–6 years, a study population very similar to the children of the ABCD cohort [20]. In addition, a mathematical model to estimate children’s energy requirements, taking growth and development into account, estimated that energy intake for 5 year old children would be approximately 1400–1600 kcal/day [49]. This corresponds to the mean parent-reported energy intake of 1530 kcal/day, which suggests that the FFQ is accurate in its estimation of the children’s energy intake, at least on a population level. Trabulsi & Schoeller proposed that the removal of implausible energy intake data from the data set could account for potential reporting error [50], but our sensitivity analysis with the exclusion of outliers did not influence the results. Therefore, we believe that the observed associations are genuine and just, but do acknowledge that potential reporting bias has important implications when attempting to associate early growth with energy intake or satiety response and thus our results should be interpreted with caution.

Third, because of the observational nature of the study with energy intake and satiety responsiveness not measured until 5 years of age, we cannot infer causality. Although the temporal order of our findings suggests an effect of early weight gain on later energy intake and satiety response, a recent study suggested reversed causality: i.e., that infants may already have an increased energy intake and diminished satiety response leading to subsequent increased infant and childhood weight gain [51]. Studies are currently underway that aim to capture feeding practices and eating behaviors from infancy onwards and relate them to future growth [52]. This will provide a better understanding of how and when energy intake and satiety response diverge between rapid infant weight gain subjects and their normal infant weight gain peers.

## Conclusions

In conclusion, accelerated infant and childhood weight gain were associated with increased energy intake and eating behavior in childhood, while rapid height gain in early infancy and childhood was associated with reduced childhood energy intake. Future studies on the biological determinants of energy intake and eating behaviors should try to further elucidate the independent associations of infant and childhood height and weight gain, aim to replicate our findings in more diverse socioeconomic and ethnic cohorts and capture energy intake and eating behaviors from an early age onwards to provide further clarification on the directionality of the associations. If future studies confirm the association between early growth and later intake and eating behaviors, these behaviors may be potential mediating factors in the association of early growth with later chronic disease risk. Then, future efforts to prevent childhood obesity through optimization of feeding patterns could in particular focus on children with accelerated infant and childhood weight gain as they are at increased risk of excessive energy intake and detrimental eating behaviors.

## Additional file

Additional file 1:
**Figure S1.** Sampling procedure of the ABCD study. **Figure S2.** Histogram showing the reported mean daily energy intake. **Table S1.** Sensitivity analysis with the exclusion of extreme energy intake values. **Table S2.** Sensitivity analysis with the exclusion of children born preterm and/or with low birth weight. (DOCX 151 kb)

## Abbreviations

ABCD: Amsterdam born children and their development
BIA: bioelectrical impedance analysis
BMI: body mass index
CEBQ: child eating behavior questionnaire
DXA: dual energy x-ray absorptiometry
FFQ: food frequency questionnaire
PA: physical activity
SES: socioeconomic status
