# Supplementary material for: The association of birth weight and postnatal growth with energy intake and eating behavior at 5 years of age – a birth cohort study
Source: Int J Behav Nutr Phys Act. 2016 Feb 4;13:15. doi: 10.1186/s12966-016-0335-4 (PMC4743237; doi:10.1186/s12966-016-0335-4)
Supplement: Additional file 1: — Figure S1. Sampling procedure of the ABCD study. Figure S2. Histogram showing the reported mean daily energy intake. Table S1. Sensitivity analysis with the exclusion of extreme energy intake values. Table S2. Sensitivity analysis with the exclusion of children born preterm and/or with low birth weight. (DOCX 151 kb) [file 12966_2016_335_MOESM1_ESM.docx]

Online Additional file to the article:

**The association of birth weight and postnatal growth with energy intake and eating behavior at 5 years of age – a birth cohort study**

Arend W. van Deutekom^1^; Mai J.M. Chinapaw^2^; Tanja G.M. Vrijkotte^3^; Reinoud J.B.J. Gemke^1^

*^1^ Department of Pediatrics, EMGO Institute for Health & Care Research, Institute for Cardiovascular Research VU, VU University Medical Center, Amsterdam, the Netherlands;*

*^2^ Department of Public and Occupational Health, EMGO institute for Health & Care Research, VU University Medical Center, Amsterdam, the Netherlands;*

*^3^ Department of Public Health, Academic Medical Centre, University of Amsterdam, Amsterdam, the Netherlands.*

# Supplementary Figure 1. Sampling procedure of the ABCD study.

**Supplementary Figure 1.** Flowchart of the sampling procedure of the ABCD cohort and selection of subjects included in this study.

# Supplementary Figure 2. Histogram showing the reported mean daily energy intake.


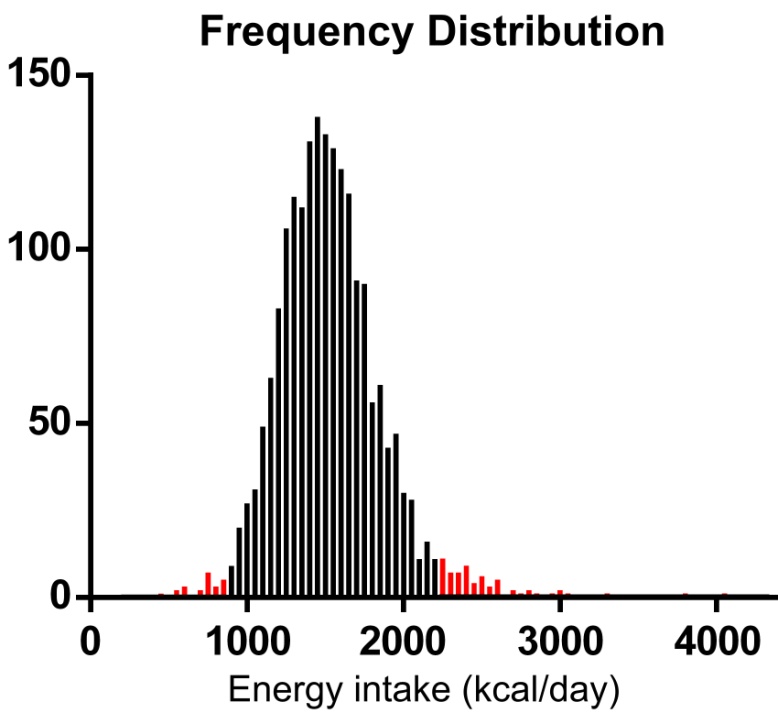


**Supplementary Figure 2.** Histogram of the distribution of the parent-reported mean daily energy intake in our cohort. In red are the values more than +2SD above the mean or less than -2SD below the mean, which are excluded in the sensitivity analysis of supplementary table 1.

# Supplementary Table 1. Sensitivity analysis with the exclusion of extreme energy intake values.

|  |  | Energy intake (kcal/day)  B (95%-CI) | Standard. Beta  (95%-CI) | P-value |
| --- | --- | --- | --- | --- |
| Weight |  |  |  |  |
| Birth weight |  | -2.7 (-23.5; 18.0) | -0,01 (-0,07; 0,05) | 0.80 |
| Conditional weight 0 – 1 mo |  | 0.01 (-17.9; 17.9) | 0,00 (-0,04; 0,04) | 1.00 |
| Conditional weight 1 – 3 mo |  | 28.4 (6.7; 50.1) | 0,09 (0,02; 0,15) | 0.01 |
| Conditional weight 3 – 6 mo |  | 25.1 (5.7; 44.5) | 0,07 (0,02; 0,13) | 0.02 |
| Conditional weight 6 – 12 mo |  | 6.4 (-13.8; 26.7) | 0,02 (-0,05; 0,09) | 0.54 |
| Conditional weight 12 mo – 5 y |  | 65.9 (22.4; 109.4) | 0,19 (0,06; 0,32) | 0.02 |
| Height |  |  |  |  |
| Conditional height 0 – 1 mo |  | -26.5 (-48.1; -2.4) | -0,08 (-0,15; -0,01) | 0.02 |
| Conditional height 1 – 3 mo |  | -36.9 (-62.4; -11.4) | -0,12 (-0,20; -0,04) | 0.005 |
| Conditional height 3 – 6 mo |  | -8.4 (-31.3; 14.5) | -0,03 (-0,10; 0,05) | 0.47 |
| Conditional height 6 – 12 mo |  | -6.8 (-30.8; 17.3) | -0,02 (-0,10; 0,06) | 0.58 |
| Conditional height 12 mo – 5 y |  | -38.3 (-76.6; 0.06) | -0,11 (-0,23; 0,00) | 0.05 |

**Supplementary Table 1.** Results of the sensitivity analysis of the association of birth weight, conditional weight and conditional height (all in Z-scores) with mean daily energy intake at age 5, with outcome values more than +2SD above the mean or less than -2SD below the mean excluded. The coefficients are presented both in the original units of measurement and in standardized betas. Analysis adjusted for sex, gestational age, ethnicity, maternal and paternal BMI, socio-economic status, smoking during pregnancy, duration of exclusive breastfeeding, current age, height and BMI, screen time and PA score at age 5.

# Supplementary Table 2. Sensitivity analysis with the exclusion of children born preterm and/or with low birth weight.

|  | Energy intake (kcal/day)  B (95%-CI) | Standard. Beta  (95%-CI) | P-value |  | Satiety Response (score)  B (95%-CI) | Standard. Beta  (95%-CI) | P-value |
| --- | --- | --- | --- | --- | --- | --- | --- |
| Weight |  |  |  |  |  |  |  |
| Birth weight | -5.9 (-30.2; 18.4) | -0,02 (0,09; 0,05) | 0.63 |  | -0.02 (-0.05; 0.01) | -0,04 (-0,10; 0,02) | 0.28 |
| Conditional weight 0 – 1 mo | 5.4 (-15.4; 26.2) | 0,01 (-0,03; 0,06) | 0.61 |  | -0.03 (-0.06; -0.00) | -0,06 (-0,12; -0,01) | 0.03 |
| Conditional weight 1 – 3 mo | 30.8 (5.5; 56.0) | 0,09 (0,02; 0,17) | 0.02 |  | -0.05 (-0.08; -0.02) | -0,08 (-0,13; -0,03) | 0.003 |
| Conditional weight 3 – 6 mo | 24.4 (2.1; 46.6) | 0,07 (0,01; 0,14) | 0.03 |  | -0.04 (-0.07; -0.01) | -0,07 (-0,12; -0,02) | 0.007 |
| Conditional weight 6 – 12 mo | 10.3 (-13.2; 33.9) | 0,03 (-0,04; 0,11) | 0.39 |  | -0.08 (-0.11; -0.05) | -0,17 (-0,24; -0,11) | <0.001 |
| Conditional weight 12 mo – 5 y | 84.3 (33.9; 134.7) | 0,24 (0,10; 0,39) | 0.001 |  | -0.13 (-0.20; -0.07) | -0,25 (-0,38; -0,13) | <0.001 |
| Height |  |  |  |  |  |  |  |
| Conditional height 0 – 1 mo | -41.7 (-66.4; -17.0) | -0,13 (-0,21; -0,05) | <0.001 |  | 0.01 (-0.02; 0.03) | -0,00 (-0,00; 0,00) | 0.61 |
| Conditional height 1 – 3 mo | -34.8 (-58.2; -11.4) | 0,11 (-0,18; -0,04) | 0.004 |  | -0.00 (-0.03; 0.02) | -0,02 (-0,60; 0,40) | 0.74 |
| Conditional height 3 – 6 mo | -7.4 (-28.6; 13.7) | -0,02 (-0,10; 0,05) | 0.49 |  | -0.03 (-0.05; 0.00) | -0,09 (-0,15; -0,00) | 0.04 |
| Conditional height 6 – 12 mo | -10.7 (-32.7; 11.4) | -0,03 (-0,10; 0,04) | 0.34 |  | -0.02 (-0.05; 0.003) | -0,04 (-0,10; 0,01) | 0.09 |
| Conditional height 12 mo – 5 y | -36.1 (-71.2; -0.97) | -0,11 (-0,21; 0,00) | 0.04 |  | -0.02 (-0.06; 0.01) | -0,04 (-0,12; 0,02) | 0.15 |

**Supplementary Table 2.** Results of the sensitivity analysis of the association of birth weight, conditional weight and conditional height (all in Z-scores) with mean daily energy intake and satiety response at age 5, with children born preterm (<37 weeks of gestation) and/or with low birth weight (birth weight more than -2SD below the mean) excluded. The coefficients are presented both in the original units of measurement and in standardized betas. Analysis adjusted for sex, gestational age, ethnicity, maternal and paternal BMI, socio-economic status, smoking during pregnancy, duration of exclusive breastfeeding, current age, height and BMI, screen time and PA score at age 5.
